# Distributed representations of the "preparatory set" in the frontal oculomotor system: a TMS study

**DOI:** 10.1186/1471-2202-9-89

**Published:** 2008-09-19

**Authors:** M Nagel, A Sprenger, R Lencer, D Kömpf, H Siebner, W Heide

**Affiliations:** 1 Department of Psychiatry and Psychotherapy, University of Luebeck, Ratzeburger Allee 160, D-23538 Luebeck, Germany; 2 Department of Neurology, University of Luebeck, Ratzeburger Allee 160, D-23538 Luebeck, Germany; 3 Department of Neurology, Christian-Albrechts-University, Schittelhelmstrasse 10, D-24105 Kiel, Germany; 4 NeuroImage-Nord, Hamburg-Kiel-Lübeck, Germany, University Medical Center Hamburg-Eppendorf, Martinistraße 52, 20246 Hamburg, Germany; 5 Department of Neurology, Celle General Hospital, Siemensplatz 4, D-29223 Celle, Germany

## Abstract

**Background:**

The generation of saccades is influenced by the level of "preparatory set activity" in cortical oculomotor areas. This preparatory activity can be examined using the gap-paradigm in which a temporal gap is introduced between the disappearance of a central fixation target and the appearance of an eccentric target.

**Methods:**

Ten healthy subjects made horizontal pro- or antisaccades in response to lateralized cues after a gap period of 200 ms. Single-pulse transcranial magnetic stimulation (TMS) was applied to the dorsolateral prefrontal cortex (DLPFC), frontal eye field (FEF), or supplementary eye field (SEF) of the right hemisphere 100 or 200 ms after the disappearance of the fixation point. Saccade latencies were measured to probe the disruptive effect of TMS on saccade preparation. In six individuals, we gave realistic sham TMS during the gap period to mimic auditory and somatosensory stimulation without stimulating the cortex.

**Results:**

TMS to DLPFC, FEF, or SEF increased the latencies of contraversive pro- and antisaccades. This TMS-induced delay of saccade initiation was particularly evident in conditions with a relatively high level of preparatory set activity: The increase in saccade latency was more pronounced at the end of the gap period and when participants prepared for prosaccades rather than antisaccades. Although the "lesion effect" of TMS was stronger with prefrontal TMS, TMS to FEF or SEF also interfered with the initiation of saccades. The delay in saccade onset induced by real TMS was not caused by non-specific effects because sham stimulation shortened the latencies of contra- and ipsiversive anti-saccades, presumably due to intersensory facilitation.

**Conclusion:**

Our results are compatible with the view that the "preparatory set" for contraversive saccades is represented in a distributed cortical network, including the contralateral DLPFC, FEF and SEF.

## Background

The neuronal processes involved in the preparation of saccadic eye movements have been intensively studied using the gap paradigm [1,2]. In this paradigm, a temporal gap of 200 ms is introduced between the disappearance of the central fixation point and the appearance of a lateral target. During the gap period, preparatory set activity gradually builds up in oculomotor areas implicated in the generation of saccades, facilitating the intention and readiness to act [3]. Invasive recordings of frontal eye field (FEF) neurons in monkeys revealed a drop in neuronal discharge at around 100 ms after the beginning of the gap period. This decrease in fixation related activity is paralleled by a slow build-up of activity in a subset of saccade neurons in the monkey's superior colliculus (SC) and frontal eye field [1,3]. Fixation disengagement and the emergence of a preparatory set facilitate the generation of saccades directed to the target (i.e. prosaccades) resulting in a shortening of the latencies of prosaccades and an increase in the amount of express saccades with latencies between 80 and 120 ms [4]. Accordingly, functional magnetic resonance imaging (fMRI) studies in humans have demonstrated an increase in task-related activity in the oculomotor system subserving saccades when a gap period was inserted before the presentation of a lateralized target [5,6]. Task-related activity in the FEF and supplementary eye field (SEF) correlated with reaction times of prosaccades during the gap-paradigm [7].

Intracortical recordings of neuronal activity in the FEF showed that the level of preparatory activity during the 200 ms gap period depends on the type of task [1,8]. When the lateralized cue and the response were spatially compatible, i.e., during a prosaccade task, saccade related neurons showed a higher prestimulus activity compared with an antisaccade task in which saccades had to be made in the direction opposite to the target [3,9]. In humans, the antisaccade task activates additional regions including the prefrontal cortices and the supramarginal gyri, relative to the prosaccade task [10]. Because the location of the cue and the direction of the saccade are decoupled, the increased neuronal activity during the antisaccade task has been attributed to the suppression of a prepotent tendency to make a saccade towards the cue and the transformation of the stimulus vector into a saccade vector away from the cue [11].

Several lines of evidence suggest that frontal oculomotor areas are involved in the generation of preparatory set activity during the gap period [12]. Single neuron studies of prosaccadic activity in non-human primates identified the FEF, SEF [13,14], and dorsolateral prefrontal cortex (DLPFC) [15-17]. These areas were also found to be activated during gap and other oculomotor paradigms in several fMRI studies [5,18-23]. The DLPFC has been implicated in saccade suppression of unwanted reflexive saccades during the antisaccade task [24] and spatial working memory [25]. Patients with lesions in the DLPFC and patients suffering from schizophrenia showed an increased amount of erroneous prosaccades during the antisaccade task, suggesting a cortical prefrontal deficit in schizophrenic patients [26,27].

Transcranial magnetic stimulation (TMS) can be used to disrupt neuronal processing at a high temporal and spatial resolution [28-30]. The disruptive impact of TMS has been successfully used to probe the functional relevance of distinct cortical areas to oculomotor control [24]. TMS of the FEF can increase the latencies of saccades to the contraversive visual hemifield or interfere with smooth pursuit eye movements [31,32]. TMS studies also showed that the DLPFC and SEF are implicated in the preparation of saccades [33-35]. For instance, single-pulse TMS of the DLPFC accelerated the initiation and increased the rate of contraversive express saccades when applied at the end of a temporal gap period lasting 200 ms [33,34,36]. Single-pulse TMS over the FEF produced a facilitatory or inhibitory effect on saccade initiation in the gap paradigm depending on the time point of TMS [37]. None of the previous studies directly compared the disruptive effects of single-pulse TMS over different frontal oculomotor areas on saccade initiation.

In this study, we applied focal TMS to the right DLPFC, FEF, and SEF to interfere with regional neuronal processing involved in the preparation of prosaccades and antisaccades. Using the gap paradigm, a temporal gap period of 200 ms was inserted between the disappearance of the central fixation point and the presentation of a lateral target. Single-pulse TMS was administered 100 and 200 ms after the fixation point had disappeared (i.e., in the middle or at the end of the gap period).

We hypothesized that TMS would delay saccade initiation in conditions with a high level of preparatory set activity: Preparatory set activity gradually builds up during the gap period. Hence, we expected TMS at target onset (i.e., at the end of the gap period) to cause stronger interference with the set activity as opposed to TMS 100 ms prior to target onset (i.e., in the middle of the gap period). The preparation of antisaccades is associated with lower preparatory set activity than the preparation of prosaccades because preparation of antisaccades concurrently involves other neuronal processes linked to saccadic suppression and vector inversion. Therefore we expected a stronger TMS-induced delay of saccade latencies for prosaccades as opposed to antisaccades [3,9]. We further hypothesized that TMS should cause a site-specific delay in saccadic response, since both regions are critically involved in the preparation of saccades [24,38]. In contrast, we predicted that TMS over the DLPFC would shorten saccade latencies through disinhibition of neuronal activity that prevents reflexive saccades [34].

## Methods

### Participants

Ten healthy right-handed male volunteers aged between 31 and 43 years (mean age: 34.4 ± 4.5 years) participated in the main experiment. Before the experiment, participants underwent standard electroencephalography (EEG) which revealed no abnormalities. We also assessed visual acuity with both eyes opened. Five participants had a visual acuity of 20/20, while the remaining participants had a visual acuity ranging between 20/25 and 20/15. Participants gave written informed consent before the experiment. The local Ethics Committee approved the study.

### Saccadic reaction time task

Participants performed two saccadic gap tasks which required participants to produce horizontal prosaccades or antisaccades in response to lateralized targets. In both gap tasks, subjects fixated a dot presented in the center of the visual field. The fixation point disappeared 200 ms before the presentation of a lateralized visual target. The fixation point was presented for a random duration of between 700 and 1300 ms. The target stimulus was a red dot which was always presented 12 degrees horizontal from the position of the fixation point in the left or right visual field. Participants were asked to maintain fixation during the gap period and react as quickly as possible to the target. All subjects could easily detect the visual target. The visual target was presented for a random duration of 1000 ms (± 300 ms). After target presentation, participants viewed a blank screen for 500 ms. New trials started with the reappearance of the central fixation point.

**Figure 1:**
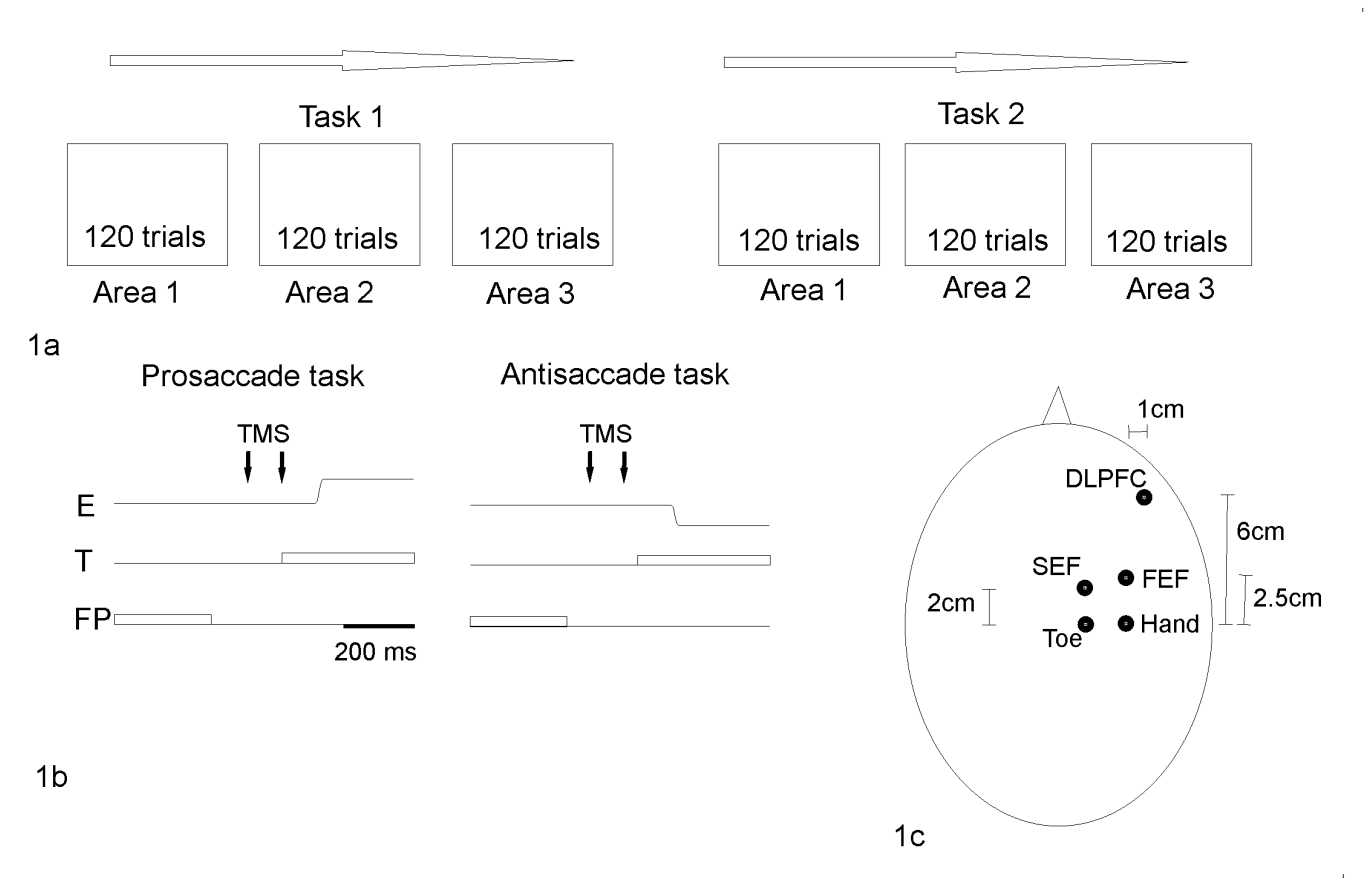
**Time line of the experiment**: **(a) **the experiment consisted of six blocks (120 trials per block). The type of task (prosaccade or antisaccade gap task) was kept constant for three consecutive blocks. Single-pulse TMS was applied to the DLPFC, FEF, and SEF in separate blocks. The order of blocks was counterbalanced across participants. **(b) **This panel illustrates the prosaccade and antisaccade gap task. E = eye movements; T = target; FP = Fixation point **(c) **Schematic drawing of the procedure to place the TMS coil over frontal oculomotor areas. Hand = Primary motor hand area; Toe = Primary motor leg area; DLPFC = dorsolateral prefrontal cortex; FEF = frontal eye field; SEF = supplementary frontal eye field.

In the first task (referred to as prosaccade gap task), participants were required to perform a saccade to the visual target as quickly as possible. In the second task (referred to as antisaccade gap task), participants were instructed to make antisaccades towards the mirror position of the target across the vertical meridian. Figure 1b illustrates the required direction of the saccade during the prosaccade and antisaccade gap task. To minimize possible effects caused by shifts in the spatial mapping rule, the saccade and antisaccade task were performed in separate blocks (see figure 1a). Subjects were trained on both tasks for approximately five minutes prior to the experiment.

Visual stimuli were presented on a 17" Sony Trinitron SE2 100 Hz color monitor triggered by the Visual Stimulus Generator (VSG II/4 Cambridge Research Systems, Rochester/UK). Participants were seated 60 cm in front of a computer screen with their head fixed on a chin and head rest. The luminance of the target (a red dot with a size of 1/2 degree) was 5 candela/m^2^, background 1/2 candela/m^2^. Eye movements were recorded at 500 Hz sampling rate by an infrared oculography Limbus-tracker (Institute of Medical Technology IMT, Dresden).

### Transcranial Magnetic Stimulation

TMS was conducted with a MagStim Rapid stimulator and a standard figure-of-eight shaped coil with a 7 cm diameter across each half-wing (Magstim Company, Spring Gardens, Wales, UK). The handle of the coil was orientated to point backwards throughout the experiment. During the task, a biphasic TMS pulse was applied either 100 or 200 ms after disappearance of the fixation point (i.e. 100 ms before presentation of the target stimulus or at the onset of target presentation). Trials with TMS were intermingled with trials without TMS in a pseudo randomized order.

In different blocks, TMS was applied over the right DLPFC, FEF, or SEF. The site of TMS was defined using the optimal site for TMS of the primary motor hand and leg area as anchor points (Figure 1c). With the coil positioned over the right primary motor hand area, we moved the coil until a single suprathreshold pulse elicited a maximum muscle twitch in the contralateral abductor pollicis brevis (APB) muscle (referred to as motor hot spot). The motor hot spot was then marked on the scalp with a pen. The same procedure was repeated over the vertex to determine the "hot spot" of the primary motor leg area using the extension of the right toe as motor response. Following the procedure described by Ro et al. [31,39], the TMS coil was moved to a position 2.5 cm anterior to the motor hot spot of the right primary motor hand area to stimulate the right FEF. The site for TMS to right DLPFC was determined according to Brandt et al. [40] and was located 6 cm anterior and 1 cm lateral to the motor hot spot of the right hand. Stimulation of SEF was performed 2 cm rostrally to the motor hotspot of the right toe [41,42].

The intensity of the stimulation was individually adjusted to 120 % of individual motor threshold (MT). MT was assessed while participants performed a slight tonic contraction of the left APB muscle at approximately 10% of maximum force. MT was defined as the intensity at which a single TMS pulse over the hot spot of the left APB muscle elicited a visible muscle twitch in the APB muscle in five out of ten consecutive trials. The MT based on a visible motor response is usually slightly higher than the MT that is determined with surface EMG [43]. No muscle twitches were elicited in the tonically contracting APB muscle when a single TMS pulse at 120% of individual MT was applied over the right FEF, DLPFC or SEF.

### Experimental design

We used a factorial design with the factors *task *(pro- or antisaccade task), *direction of the saccade *(ipsiversive (to the right) or contraversive (to the left) to TMS), *site of TMS *(right DLPFC, right FEF, or right SEF) and *type of TMS *(no TMS, TMS in the middle of the temporal gap period or TMS at the end of the gap).

The experiment consisted of six experimental blocks. Participants performed one block per task and TMS site. The order of experimental blocks was counterbalanced across participants. Within each block, the site of TMS and the task were kept constant, while the TMS condition and target location were pseudorandomly intermingled. Each experimental block consisted of 120 trials (20 trials per experimental condition), lasting 330 s. The total experiment lasted for approximately 60 minutes.

### Control experiment

The control experiment was motivated by previous TMS studies showing that the auditory and somatosensory stimulation can influence response times [44,45]. For instance, responses are speeded up when TMS is applied at approximately the same time as the instruction cue [44,46]. This non-specific effect of TMS has been attributed to intersensory response facilitation [47].

Six subjects participated in the control experiment (mean age: 33.8 ± 4.4 years). Three subjects had already participated in the main experiment. We used a 'realistic' sham procedure reported by Okabe [48] to match as closely as possible the peripheral (auditory and sensory) stimulation caused by TMS. This procedure has been successfully used in other TMS studies [48-50]. The sham procedure consisted of ineffective single-pulse TMS and concurrent electrical stimulation of the scalp. Ineffective TMS was performed with a standard figure-of-eight coil placed over the vertex. The coil was vertically tilted and only the outer margin touched the scalp. Electrical stimuli were applied using a Bravo Nicolet EMG device (Nicolet Biomedical, Offenbach, Germany) and bipolar electrodes placed over the C4 position of the international 10 – 20 system for EEG electrode placement. The electrical stimulus had a square wave configuration (0.1 ms duration). The amplitude was set at 0.6 mA, which was always above individual perceptive threshold. The sensation induced by electrical stimulation was reported to be similar compared to real single-pulse TMS. Otherwise, experimental procedures were identical to the main experiment. Compared to stimulation with a sham coil, our realistic sham procedure has the advantage that the electrical stimulation of the scalp mimics the cutaneous sensation evoked during real TMS [48,50,49]. Accurate sham procedures are important because it has been found that some sham TMS conditions produce substantial cortical stimulation in the monkey brain, yet merely tilting the coil to 90° eliminates such effects [51].

### Data analysis

Saccade latencies were determined on a trial-by-trial basis with a semi-automatic MatLab routine (MatLab 6.5R13^©^, The Math Works Inc., Natick, Mass., USA). Saccades were identified automatically if the initial eye velocity was higher than 30°/s, amplitude > 1°, and duration > 10 ms. Eye-blinks were identified and excluded on the basis of characteristic appearance in eye position and velocity charts. Saccades with shorter latencies than 80 ms were categorized as anticipatory saccades. Anticipatory saccades and saccades with latencies longer than 400 ms were excluded from further analysis.

#### Prosaccades

Saccades were defined as prosaccades when the participant performed a primary saccade towards the target with a latency of > 120 ms. The rate of express saccades with latencies between 80 and 120 ms induced by the gap paradigm was analyzed separately [34]. Directional errors of the prosaccades were not considered.

#### Antisaccades

Saccades were only classified as antisaccades if the subject performed a primary saccade in the direction opposite to the peripheral target. The antisaccade latency was defined in the same way as the prosaccade latency. In each participant, we also calculated the error rate for each experimental condition. An antisaccade was classified as erroneous if a participant made a primary saccade towards the visual target. The error rate was expressed as a percentage of the total number of saccadic responses.

In each participant, we calculated the mean saccade latency for each experimental condition. We were mainly interested in the effect of TMS on saccade latencies. To reduce inter-individual variability in overall RTs, we calculated the difference in saccade latency during trials with TMS compared with trials without TMS in each subject.

In addition to saccade latency as a measure of saccade initiation, we also tested for TMS induced changes in saccade execution. To this end, we calculated the *duration, peak velocity *and *gain *of all pro- and antisaccades that were included in the data analysis.

### Statistical analyses

Statistical analysis was performed using SPSS^© ^software (Chicago, Illinois, USA). Since we were mainly interested in the effects of TMS on saccade latencies, we used the change in saccade latency associated with TMS as a dependent variable for statistical analysis. We first conducted a four-factorial (within-subject) repeated measure ANOVA including the factors *task *(pro- and antisaccade task), *direction *of horizontal saccade (ipsiversive and contraversive), *timing of TMS *(TMS at 100 and 200 ms after gap period onset) and *site *of TMS (FEF, DLPFC and SEF). Because the four-factorial ANOVA showed that TMS had a different effect on the latencies of pro and antisaccades (main effect of *task*), we computed separate follow-up ANOVAs for each saccadic task. These follow-up ANOVAs included the factors *direction *of horizontal saccade (ipsiversive and contraversive), *timing of TMS *(TMS at 100 and 200 ms after gap period onset) and *site *of TMS (FEF, DLPFC and SEF).

To analyze how the experimental factors influenced the frequency of express and error saccades, we calculated the frequency of express and error saccades for each experimental condition. Using the rate of express saccades or error saccades as dependent variable, we performed three-factorial ANOVAs to assess differences in the frequency of express saccades for the prosaccade and error-saccades for the antisaccade task. Because express saccades mainly occurred during the prosaccade task, the TMS-induced changes in express saccades were only explored during the prosaccade task. The effect of TMS on error saccades was only examined in the antisaccade task because error rates were very low in the prosaccade task. The ANOVA model included the factors, *direction *of saccade (ipsiversive and contraversive), *timing of TMS *(TMS at 100 and 200 ms after gap period onset) and *site *of TMS (FEF, DLPFC and SEF).

Statistical analyses of the control experiment was based on an ANOVA model that included the factors *task *(pro- and antisaccade),*direction of saccade *(ipsiversive and contraversive) and *time of electric stimulation *(electric stimulation at 100 and 200 ms after gap period onset).

Conditional to a significant F-value, post hoc t-tests were performed to explore the strength of main effects and the patterns of interaction between experimental factors. Statistical threshold was set at P < 0.05. The Greenhouse-Geisser method was used to correct for non-sphericity. All data are given as mean ± SD.

## Results

None of the participants reported adverse effects. Participants made anticipatory eye movements prior to the appearance of the cue in 2.6% (range: 0 – 7.4%) of the trials during the prosaccade task and in 0.9% (range: 0 – 5.7%) during the antisaccade task. Saccades with latencies shorter than 80 ms were made on average in 6.38% (range: 0 – 23.0%) of trials in the prosaccade task and in 4.0% (range: 0 – 14.4%) of the trials in the antisaccade task. These trials were excluded from further analysis.

### Change in saccade latency

**Figure 2:**
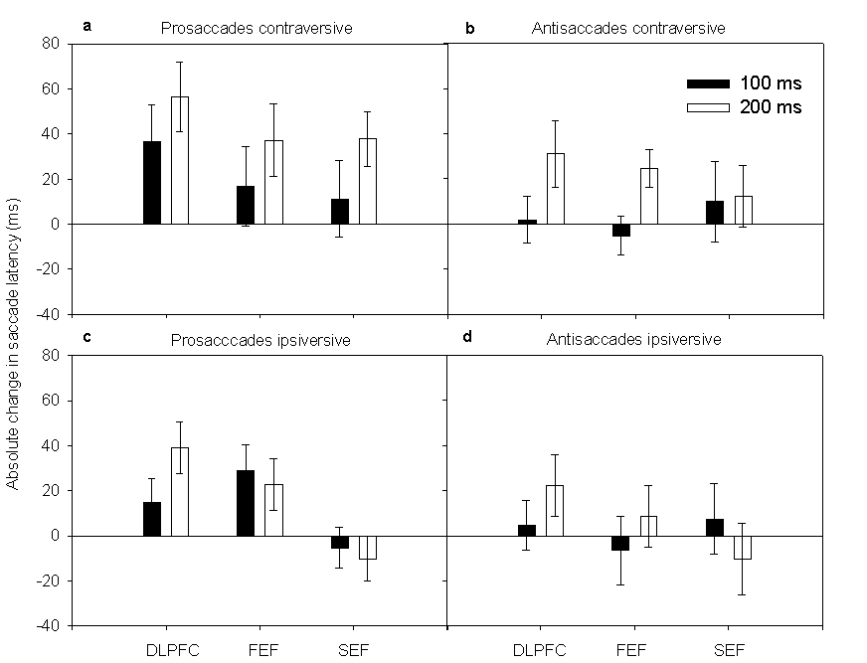
**Latency change of saccades**: Mean change of (± SEM) latencies; ipsiversive or contraversive prosaccades **(a, c) **and antisaccades **(b, d) **for each site of TMS.

Table 1 lists the mean latencies of the saccades during the prosaccade and antisaccade task. The changes in saccade latencies relative to the condition without TMS are illustrated in Figure 2. Using the difference in RTs as dependent variable, the four-factorial ANOVA showed a main effect of *task *(F_(1, 9) _= 137.9; p < 0.0001). Figure 2 shows that this was caused by a stronger TMS-induced increase in saccade latency with prosaccades than with antisaccades. The main effect of site of stimulation (F_(1.7, 15.3) _= 4.1; p = 0.060) and time of TMS (F_(1, 9) _= 4.6; p = 0.069) were statistically not significant. TMS to the DLPFC tended to increase saccade latencies more than TMS to SEF during both conditions. Furthermore, TMS at the end of the gap period onset tended to induce a stronger delay of saccade onset than TMS in the middle of the gap.

**Table 1:** Mean latencies (± SD) of ipsi- and contraversive pro- and antisaccades for each TMS site.

Prosaccades		NO TMS	TMS 100 ms	TMS 200 ms
DLPFC	contraversive	172 (26)	208 (46)	228 (54)
	ipsiversive	173 (34)	213 (28)	188 (36)
FEF	contraversive	187 (35)	203 (47)	224 (43)
	ipsiversive	160 (15)	183 (29)	189 (41)
SEF	contraversive	181 (33)	193 (44)	219 (32)
	ipsiversive	178 (34)	168 (25)	172 (20)

Antisaccades		NO TMS	TMS 100 ms	TMS 200 ms

DLPFC	contraversive	243 (25)	249 (21)	278 (40)
	ipsiversive	268 (33)	277 (24)	295 (27)
FEF	contraversive	239 (17)	234 (26)	264 (24)
	ipsiversive	250 (26)	243 (37)	259 (38)
SEF	contraversive	252 (37)	261 (32)	264 (26)
	ipsiversive	262 (38)	270 (48)	252 (42)

There was also a significant interaction between the direction of the saccade and the type of task which was due to a stronger increase in RTs for contraversive saccades during the prosaccade task (F_(1, 9) _= 28.4; p = 0.002). The latencies of prosaccades were increased to a greater extent when TMS was applied over DLPFC at the end of the gap period. This was reflected by an interaction between the factors site of stimulation, time of TMS and type of task (F_(2.5, 22.9) _= 3.5; p = 0.041).

Following-up on this analysis, we performed two three-factorial ANOVAs to separately test how TMS influenced saccade latencies during the prosaccade and antisaccade task. This ANOVA model included the within subject factors *direction *of horizontal saccade (ipsiversive and contraversive), *timing *of TMS (TMS at 100 and 200 ms after gap period onset) and *site *of TMS (FEF, DLPFC and SEF).

### Prosaccades

**Figure 3:**
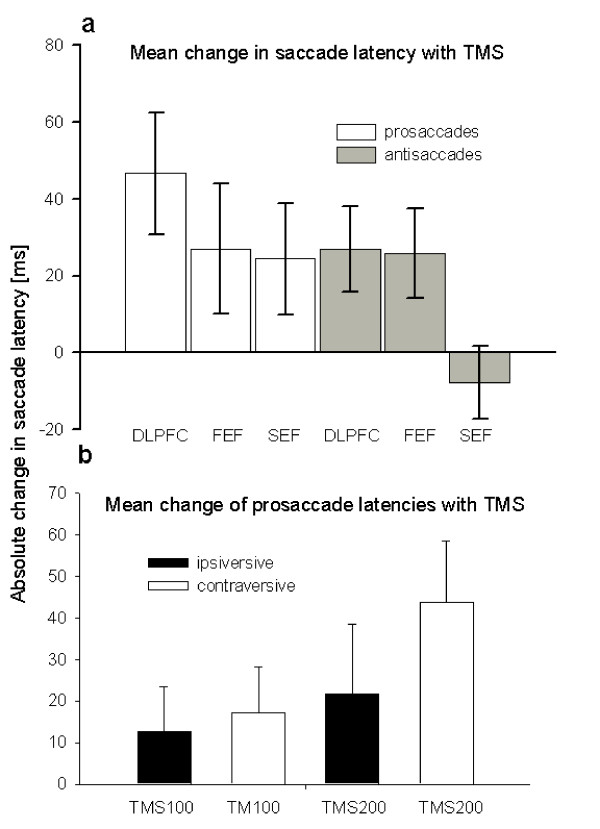
**(a) Overall mean change (± SEM) in saccade latencies**: the increase of saccadic latencies induced by TMS to the DLPFC, FEF, and SEF during the prosaccade and antisaccade task relative to trials without any TMS. Mean latencies of ipsi- and contraversive prosaccades and trials with TMS at 100 and 200 ms after gap period onset are pooled together to demonstrate the main effect of the site of TMS. **(b) **Changes (mean ± SEM) in saccade latencies of ipsiversive and contraversive prosaccades caused by TMS applied at 100 and 200 ms after gap period onset. The data obtained with TMS over DLPFC, FEF, and SEF are pooled together to illustrate the overall influence of timing of TMS on saccade latencies.

Using the TMS-induced difference in saccade latency as dependent variable, a three-factorial repeated measures ANOVA revealed a main effect of *time *of TMS with prosaccades (F_(1, 9) _= 15.9; p = 0.004). The main effect of time was caused by a stronger increase in saccadic latencies with TMS at 200 ms compared to TMS at 100 ms (*t *= 2.3, p = 0.046). The interaction between direction of saccade and time of TMS was also significant (F_(1, 9) _= 14.5; p = 0.005). TMS produced a stronger increase in prosaccade latencies towards contraversive targets in the left hemifield as opposed to prosaccade latencies towards ipsiversive targets in the right hemifield when applied at the end rather than in the middle of the gap period (Figure 2 and Figure 3b). There was a main effect of the *site *of stimulation (F_(1.8, 16.4) _= 4.4; p = 0.030; Figure 3a). This was caused by a stronger delay in saccadic RTs during DLPFC stimulation relative to stimulation of FEF (*t *= 2.3, p = 0.04) or SEF (*t *= 3.1, p = 0.01).

### Antisaccades

In the antisaccade task, three-factorial repeated measures ANOVA revealed no main effect of *time *or *direction *of saccade on the TMS-induced change in saccade latency. Also the *site *of stimulation did not reach significance (F_(1.7, 15.7) _= 3.65; p = 0.098). In contrast to TMS over the SEF, TMS of the DLPFC and FEF caused an increase in saccadic latency at the end of the gap period relative to TMS in the middle of the gap. This was reflected by an interaction between *time *of TMS and *site *of stimulation (F_(1.6, 16.4) _= 4.97; p = 0.037). The interaction between the saccade direction and the time of TMS was also significant (F_(1, 9) _= 5.68; p = 0.049). TMS had a stronger effect on latencies of contraversive saccades when applied 200 ms after the disappearance of the fixation cue (Figure 2).

### Error saccades during the antisaccade task

Repeated measures ANOVA revealed no main effect of *saccade direction*, *timing *of TMS or *site *of TMS and no interaction among these factors (p > 0.08). After pooling the error rates during TMS over the DLPFC, FEF, and SEF together, mean error rate was 21% (ipsiversive (right directed) saccades: 19%, range: 8 – 32%; contraversive (left directed) saccades: 23%, range: 17 – 27%) in trials without TMS.

### Express saccades during the prosaccade task

Repeated measures ANOVA showed no main effect of *saccade direction*, *timing *of TMS or *site *of TMS and no interaction among these factors (p > 0.08). In particular, there was no difference in the rate of express saccades between ipsiversive and contraversive saccades. In trials in which no TMS was applied, the mean rate of express latencies was 6.5% (ipsiversive saccades: 5.0%, range, 0 – 22.5%; contraversive saccades: 7.7%, range: 0–26.8%).

### No effects of TMS on mean saccade duration, peak velocity and gain

Including the duration of the pro and antisaccades in two separate 3-factorial repeated measures ANOVA's revealed no significant main effects of *time*, *direction *or *site *(p > 0.13). Interactions were also not significant (p > 0.07). Analysis of the peak velocity of the saccades revealed no main effect of *time*, *direction*, *site *(p > 0.23) or interaction (p > 0.22) among these factors. Also the analysis of the gain revealed no main effect or interaction (main effect: p > 0.09, interaction: p > 0.18). Mean duration of prosaccades: 49 ms (SEM: 1.98); antisaccades: 55 ms (SEM: 3.7). Mean peak velocity of prosaccades: 393.7°/s (SEM: 17.6); mean peak velocity of antisaccades: 333.9°/s (SEM: 32). Mean gain of prosaccades: 0.93 (SEM: 0.036); mean gain of antisaccades: 0.91 (SEM: 0.08).

### Control experiment

**Figure 4:**
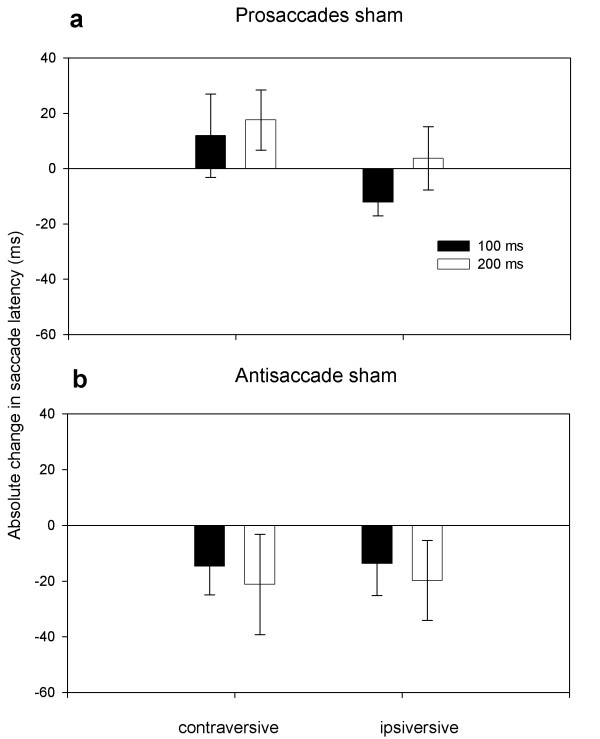
**Mean Change (± SEM) of saccadic latencies**: prosaccades (a) and antisaccades (b) with sham stimulation relative to baseline without sham stimulation.

Figure 4 illustrates the changes in saccade latencies during realistic sham stimulation relative to baseline without stimulation. Sham stimulation tended to shorten the latencies of contraversive and ipsiversive antisaccades, but was statistically not significant. In the prosaccade task, the latencies of contraversive prosaccades were slightly but not significantly prolonged while the latencies of ipsiversive prosaccades were not affected by sham stimulation. The differential effect of sham stimulation on the prosaccade and antisaccade task was confirmed by a main effect of *task *in the ANOVA (F_(1, 5) _= 17.84; p = 0.013). The interaction between the *direction *of saccade and the *type of task *(F_(1, 5) _= 7.19; p = 0.05) was not significant. The factor *time *of sham stimulation (F_(1, 5) _= 6.22; p = 0.052), the interaction between *time *of sham stimulation and *type *of task (F_(1, 5) _= 5.35; p = 0.059) and the interaction between the *direction *of saccade and the *type of task *(F_(1, 5) _= 7.19; p = 0.05) was not significant. There was no interaction among all three factors (F_(1, 5) _= 0.30; p = 0.75). Moreover we found no significant difference between the latencies of trials without TMS during the TMS experiment and trials without electric stimulation during the sham control experiment (p > 0.074).

Follow-up ANOVAs showed no main effects or interactions between the saccade direction and the time of TMS application when changes in saccade latencies were examined separately for the prosaccade or antisaccade task (p > 0.204).

## Discussion

We found that single-pulse TMS over right DLPFC, FEF, and SEF delayed the initiation of contraversive saccades in the context of the saccadic gap paradigm. The delay in saccade latency was most pronounced when subjects made prosaccades as opposed to antisaccades. In the prosaccade task, the TMS-induced delay was stronger when the TMS pulse was applied at the end of the gap period, i.e., at target onset, rather than in the middle of the gap period. This was particularly apparent during contraversive prosaccades. Although TMS at all three frontal sites affected saccade latencies, TMS of the right DLPFC was more effective at delaying the onset of prosaccades than stimulation of FEF and SEF. In contrast, realistic sham TMS tended to decrease the latencies of antisaccades and to shorten the latencies of prosaccades. We discuss the implications of the present findings in the context of current concepts on preparatory set activity in the oculomotor network.

Focal TMS to the DLPFC, FEF and SEF produced a consistent delay in saccade latencies, especially in the prosaccade task. This finding does not support the concept that a single frontal oculomotor area is exclusively involved in building up the preparatory set during the gap period before target appearance. It rather suggests that a distributed set of oculomotor regions sustains the preparatory set, including the DLPFC, FEF, and SEF. This view is in agreement with brain mapping studies which demonstrated a tight coupling of functional activity in the SEF, FEF and DLPFC during the generation and suppression of saccades [7,20,52-54].

If preparatory set activity is represented in a distributed network of oculomotor regions, one might expect that focal TMS during the gap period should not interfere with saccade generation because the focal lesion should be compensated by those nodes of the network that were not targeted with TMS. The fact that focal TMS to DLPFC, FEF, and SEF delayed the onset of saccades raises the possibility that the generation of preparatory set relies on the optimal integration of neuronal activity across the oculomotor network rather than on the local activity of a distinct area. It is worth to point out that the regional neuronal excitation induced by TMS can transsynaptically spread to remote areas via cortico-cortical and cortico-subcortical axons that connect the stimulated area with other nodes of the network [55-57]. Hence, TMS may have interfered with saccade generation by effectively perturbing the functional integration of distributed preparatory set activity within the oculomotor system.

Since we only probed oculomotor function at a behavioural level, we can only speculate about the underlying neurobiological mechanisms that mediated the delay in saccade latency. One possible mechanism that mediated the lesion effect is that TMS disrupted the cortico-cortical cross-talk among the stimulated cortical premotor areas. Alternatively, TMS may have disrupted neuronal activity of cortico-subcortical projections to the superior colliculus [33,34,36]. Such a mechanism is biologically plausible as invasive recordings from the FEF in primates which identified preparatory set activity in cortico-subcortical neurons that directly project onto the superior colliculus [3]. Pharmacological inactivation of the superior colliculus has previously bee shown to decrease saccadic velocities and reduce express saccades [58]. Such changes were absent in the present study. Therefore, it is unlikely that a cortico-subcortical spread of excitation from the stimulated cortex to the superior colliculus did not make a major contribution to the disruptive effect of TMS. Finally, a cortico-cortical spread of excitation from DLPFC, FEF, or SEF to a remote cortical area (i.e. the posterior parietal eye field) might have disrupted neuronal activity in that area, causing the disruptive effect of TMS on saccade initiation [12,24].

The spread of TMS-induced excitation to connected cortical areas will be facilitated if the excitability of the cortico-cortical interneurons is high. Preparatory set activity gradually builds up during the temporal gap period that separates the disappearance of the fixation point and the appearance of the lateral target [11]. The pre-stimulus neuronal activity immediately before stimulus presentation during the gap period of 200 ms influenced the latency of contraversive saccades. The higher the activity the shorter the subsequent latency [9]. As a consequence, the excitability within the oculomotor network and its connections gradually increase towards the end of the gap.

We hypothesized that the TMS-induced lesion effect should be strongest at the end of the gap period because the neuronal activity induced by the TMS pulse can easily spread throughout the activated network and disrupt the inter-regional integration of preparatory set activity at the systems level. In accord with our prediction, TMS was most effective in delaying saccade latencies when the magnetic pulse was applied at the end of the gap period when preparatory set activity had reached its maximum. We conclude that focal TMS to a single node of the frontal oculomotor network can effectively reset preparatory set activity to a lower level, and the "resetting" of preparatory set activity is most effective at a high level of preparation.

The same line of reasoning applies to the observation that TMS produced a longer delay in saccade onset during the prosaccade task, especially when participants made contraversive prosaccades. As outlined in the introduction, preparatory set activity is more pronounced with prosaccades as opposed to antisaccades [3,9]. Because stimulus-response mapping was dissociated, additional cognitive processes such as suppression of reflexive prosaccades towards the target and vector inversion became relevant during the preparation of antisaccades [10,59]. These competing processes, limited the build-up of preparatory set activity [1]. Therefore, we attribute the stronger delay in saccade latency with prosaccades to a stronger expression of preparatory set activity in the oculomotor system during the prosaccade task, rendering the oculomotor network more susceptible to focal single-pulse TMS.

The strongest delay in saccade latency was evoked with TMS over the DLPFC, emphasising a prominent role of the DLPFC in the preparation of saccades after a short gap period [17]. The DLPFC has been implicated in top down control, monitoring and intention during preparation of saccades [17,60,61]. The connectivity pattern of the DLPFC supports a central role of the DLPFC in the preparation and selection of saccades as the DLPFC has dense anatomical connections to a broad amount of cortical and subcortical regions, including the superior colliculi, FEF, SEF, and the parietal eye field [17,60,61]. However, a note of caution is warranted when one directly compares the "magnitude" of a TMS-induced lesion effect between different cortical areas. Although we used the same stimulation coil and stimulus intensity, it is possible that the 'lesion effect' induced over the DLPFC was stronger than the lesion effect produced by TMS of the FEF and SEF. Since the FEF is located in the depth of the caudal tail of the superior frontal sulcus and the SEF is buried in the interhemispheric fissure, TMS of the SEF or FEF might have required higher stimulus intensity than TMS to the DLPFC to be equally disruptive. If so, TMS of the FEF and SEF may produce the same magnitude of the "lesion effect" on saccadic latency in the gap paradigm if TMS is applied at a higher intensity.

In the present study, focal TMS over the DLPFC delayed the initiation of prosaccades without changing the rate of express saccades. Unlike in the present study, previous studies reported that TMS to the DLPFC at the end of a 200-ms gap period single-pulse TMS shortened the latency of prosaccades [33,34,36] along with an increased rate of express and error saccades during the antisaccade task [33,34,36,62]. A reduction in the initiation of prosaccades was also observed in the gap paradigm when single-pulse TMS was applied at target onset over the right FEF [37]. In these studies, it was proposed that TMS disrupted neuronal activity suppressing reflexive prosaccades, thereby facilitating saccade initiation. This interpretation ties in with lesion and fMRI studies that used the antisaccade paradigm showing that the DLPFC is involved in inhibition of unwanted reflexive prosaccades [17].

The differences in the behavioural effects between the present study and previous work may be related to differences in experimental design. Since we were mainly interested in the preparation of externally cued saccades, we chose an oculomotor paradigm with a constant response rule based on the presentation of a single target. Prosaccade and antisaccade tasks were tested in separate blocks to minimize the influence of switches between two tasks.

Previous studies used a wide range of saccadic paradigms, including gap-and-overlap paradigm [37], gap and no-gap paradigms [34] or paradigms with more target locations and different colours or target shapes [63]. Sometimes, different types of paradigms were intermingled [34,37] so that participants had to switch between different tasks. In these tasks, performance relies on additional neuronal processes such as response inhibition, spatial attention and decision making or spatial working memory which may account for the different behavioural effects of focal TMS on saccadic latencies.

While focal TMS of the DLPFC, FEF, and SEF interfered with the initiation of prosaccades and antisaccades, TMS had no influence on the duration, peak velocity, or gain of saccades) in the context of a preparatory set. Other studies have shown that TMS of the FEF can effectively disrupt the execution of spatially cued saccades without a preceding gap [64,65]. Again, this shows that the disruptive effect of TMS on oculomotor control critically depends on the experimental task during which TMS is applied.

There are also some technical differences regarding TMS which may contribute to the apparently discrepant results. While previous studies placed the TMS coil 5 cm anterior to the motor hand area to target the DLPFC [33,34,36], we centred the coil 6 cm anterior and 1 cm lateral to the motor hand area according to Brandt et al. [40]. There is converging evidence for a functional segregation within the DLPFC in humans and monkeys [66,67]. Therefore, it is possible that in previous studies, prefrontal TMS preferentially targeted a site that is more relevant to the suppression of reflexive saccades (i.e., mid dorsolateral prefrontal cortex) whereas in the present study, TMS over DLPFC may have stimulated a prefrontal site that is more specialised in generating and maintaining preparatory set activity.

Several studies showed that the FEF can influence visual processing in the occipital cortex [68-73]. Olk et al. [68] found evidence for a TMS effect on visual processing during a task requiring saccadic suppression before pro and antisaccades. Early influence on visual response modulation both before and after target presentation in the occipital cortex lends further support to the concept of an attentional top-down influence on early visual processing [74,75]. Of note, single-pulse TMS of the FEF affected selective visual processing in occipital cortex during the initiation of a spatially cued saccade, presumably by disrupting fronto-occipital feedback connections [69-72,76,77]. If the distributed preparatory set activity in frontal oculomotor areas enhanced visual processing in the occipital cortex via fronto-occipital projections, frontal TMS might have disrupted this attentional enhancement of visual processing, resulting in a delay of saccade initiation.

Finally, non-specific TMS effects caused by auditory and somatosensory stimulation need to be considered when interpreting the TMS-induced changes on response latencies [44,78,79]. On the one hand, saccadic latencies may be shortened by intersensory facilitation if TMS is applied with the onset of the target cue (for review see: Nickerson [47]). This might be of relevance to the present results because the most prominent changes in saccadic latencies occurred when real TMS was applied at the onset of target presentation. To assess the non-specific TMS effects on task performance, we conducted a control experiment using a realistic sham procedure. Overall, the effects of sham stimulation were smaller in magnitude than the effects found with real TMS. Interestingly, sham stimulation had a differential effect on the initiation of prosaccades and antisaccades. The latencies of contraversive and ipsiversive antisaccades were slightly shortened. The onset of contraversive (but not ipsiversive) prosaccades was slightly prolonged by sham stimulation. The facilitatory effect of realistic sham TMS on the initiation of antisaccades is not surprising because auditory and somatosensory cues to visual targets have been shown to reduce the latencies of antisaccades in the context of a gap paradigm [80,81]. A tentative interpretation is that with realistic sham-TMS, intersensory facilitation prevailed in the antisaccade task, whereas the response bias caused by lateralized cueing was prevalent in the prosaccade task. Somatosensory and auditory stimuli during the gap are less efficient in reducing the prosaccadic latencies because the gap effect is mainly based on visual fixation disengagement [82].

A direct statistical comparison of the results obtained with real TMS and realistic sham TMS was not possible because only three out of six individuals who participated in the control experiment also had participated in the main experiment. Therefore, we can only descriptively compare the behavioural effects of real TMS over frontal areas with those induced by realistic sham TMS. The observed changes in saccadic latencies with sham stimulation differed in magnitude and pattern from those induced by TMS over DLPFC, FEF and SEF. The discrepant effects of real and sham TMS point to a specific effect of real TMS on the initiation of saccades. However, we cannot exclude the possibility that non-specific TMS have modified the behavioural effects that were directly caused by transcranial brain stimulation. For instance, real TMS might have produced a longer delay of saccade onset in the antisaccade task, but the full magnitude of the "lesion" effect was attenuated by the acceleratory effects of intersensory facilitation.

### Methodological considerations

Frameless stereotaxy is increasingly used to place the coil over the cortical target area, because neuronavigated TMS enables a more precise placement of the coil over the target area. Here the motor cortex was used as an anchor point to define the sites of stimulation. This approach has been successfully used in previous TMS studies on oculomotor control to target the DLPFC, FEF and SEF [31,39-42,62,83,84]. The DLPFC in particular exhibits a broad anatomical variability and therefore demands a more careful interpretation of the DLPFC related findings [85]. Yet suboptimal targeting of frontal oculomotor areas might have led to an underestimation of the effects of TMS on saccade latency, especially with TMS of the DLPFC.

We chose a stimulus intensity of 120% of active motor threshold for TMS. MT was determined by visual inspection of the TMS induced muscle twitch in the left APB muscle. We assumed that this intensity would be sufficiently high to produce a "lesion effect" in the frontal eye field which is buried in the depth of the superior frontal sulcus and in the supplementary eye field in the interhemispheric fissure. It may be argued that a relatively high intensity of TMS reduced the focality of TMS causing direct spread of excitation to other oculomotor areas. However, this is unlikely as previous TMS studies of oculomotor control used the same or even higher stimulation intensities (e.g., 80 – 90% of maximal stimulator output) and still produced regional specific effects on the initiation of eye or finger movements [41,42,62,86].

It would have been preferable to include the realistic sham procedure already in the experimental design of the main experiment. However, this would have significantly prolonged the duration of the experiment, and may have adversely affected performance due to fatigue. It should also be noted that site specific effects of TMS on saccade latency, effective TMS of other brain regions during the main experiment already provided an appropriate control condition. An advantage of the sham procedure relative to previously used sham conditions [62,84,87] was that the sham procedure mimicked somatosensory and auditory stimulation without stimulating brain tissue. Therefore, we think that our control experiment was sufficient to characterize the non-specific effects of TMS on saccade latencies in the gap paradigm.

## Conclusion

We conclude that the DLPFC, FEF and the SEF of the right hemisphere are involved in generating the preparatory set activity of saccades directed contraversive to TMS during the gap paradigm. A functional "lesion" of a single cortical node of the network of the right hemisphere can affect saccade initiation, presumably by transiently reducing the level of preparatory set activity of contraversive to TMS directed saccades. The TMS-induced delay of these saccades is particularly prominent if "preparatory set" activity within right-hemispheric frontal oculomotor areas is high.

## Authors' contributions

AS and MN carried out all experiments and analyzed the data, WH and DK provided infrastructure support for the experiments and critically reviewed the manuscript. WH, AS and MN designed the study. HS, MN and RL wrote and reviewed the manuscript. All authors read and approved the final manuscript.
